# Emerging Tick-borne Infections in the Upper Midwest and Northeast United States Among Patients With Suspected Anaplasmosis

**DOI:** 10.1093/ofid/ofae149

**Published:** 2024-03-15

**Authors:** Megan E Reller, Emily G Clemens, Johan S Bakken, J Stephen Dumler

**Affiliations:** Division of Infectious Diseases, Department of Medicine, Johns Hopkins University School of Medicine, Baltimore, Maryland, USA; Division of Infectious Diseases, Department of Medicine, Johns Hopkins University School of Medicine, Baltimore, Maryland, USA; St Luke's Hospital, Duluth, Minnesota, USA; Department of Pathology, Uniformed Services University of the Health Sciences, Bethesda, Maryland, USA; St Luke's Hospital, Duluth, Minnesota, USA; Department of Pathology, Uniformed Services University of the Health Sciences, Bethesda, Maryland, USA

**Keywords:** *Babesia*, *Borrelia*, *Ehrlichia*, *Rickettsia*, tick-borne disease

## Abstract

**Background:**

Emerging tick-transmitted illnesses are increasingly recognized in the United States (US). To identify multiple potential tick-borne pathogens in patients from the Upper Midwest and Northeast US with suspected anaplasmosis, we used state-of-the-art methods (polymerase chain reaction [PCR] and paired serology) to test samples from patients in whom anaplasmosis had been excluded.

**Methods:**

Five hundred sixty-eight patients without anaplasmosis had optimal samples available for confirmation of alternative tick-borne pathogens, including PCR and/or paired serology (acute-convalescent interval ≤42 days).

**Results:**

Among 266 paired serology evaluations, for which the median acute-convalescent sampling interval was 28 (interquartile range, 21–33) days, we identified 35 acute/recent infections (24 [9%] *Borrelia burgdorferi*; 6 [2%] *Ehrlichia chaffeensis/Ehrlichia muris* subsp *eauclairensis* [*EC/EME*]; 3 [1%] spotted fever group rickettsioses [SFGR], and 2 [<1%] *Babesia microti*) in 33 (12%) patients. Two had concurrent or closely sequential infections (1 *B burgdorferi* and *EC/EME*, and 1 *B burgdorferi* and SFGR). Using multiplex PCR and reverse-transcription PCR, we identified 7 acute infections (5/334 [1%] *Borrelia miyamotoi* and 2/334 [1%] *B microti*) in 5 (1%) patients, including 2 with *B microti*–*B miyamotoi* coinfection, but no *Borrelia mayonii*, SFGR, *Candidatus* Anaplasma capra, Heartland virus, or Powassan virus infections. Thus, among 568 patients with ruled-out anaplasmosis, 38 (6.7%) had ≥1 agent of tick-borne illness identified, with 33 patients (35 infections) diagnosed by paired serology and 5 additional patients (7 infections) by PCR.

**Conclusions:**

By identifying other tick-borne agents in patients in whom anaplasmosis had been excluded, we demonstrate that emerging tick-borne infections will be identified if specifically sought.

## Teaser

By conducting state-of-the-science tests to identify other tick-borne agents in patients in whom anaplasmosis had been excluded, we demonstrate that emerging tick-borne infections will be identified if specifically sought.

First identified in 1990, *Anaplasma phagocytophilum* causes human granulocytic anaplasmosis (HGA or anaplasmosis), presents as a nonspecific acute febrile illness, and is transmitted by the deer or black-legged tick *Ixodes scapularis* [1]. *Ixodes scapularis*, the most common tick vector in the United States (US), causes tick-borne diseases in the Upper Midwest (UM) and Northeast (NE) US. However, novel agents, changing epidemiologic patterns, and additional vectors have been discovered when explicitly sought. We report a range of tick-borne illnesses that mimic anaplasmosis when suspected.

## MATERIALS AND METHODS

### Patients and Samples

We studied archived specimens from 568 patients residing in the Upper Midwest (UM) and Northeast (NE) US who presented May 1991–July 2009 with suspected anaplasmosis. Although additional clinical and laboratory information was provided for a subset of patients (85 from the UM), etiologic diagnoses were not. Specimens included 266 paired sera for antibody testing and 343 whole blood specimens for polymerase chain reaction (PCR) analysis. We excluded those with *A phagocytophilum* infection confirmed by ≥1 Centers for Disease Control and Prevention (CDC) criteria: 4-fold immunoglobulin G (IgG) titer rise by indirect fluorescent antibody (IFA) between acute and convalescent (paired) sera, PCR positive, isolation from blood, and acute-phase blood smear positive [2]. We used available sera and DNA from blood (archived at −20°C) and/or ethylenediaminetetraacetic acid (EDTA)–anticoagulated blood (preserved at −80°C) to diagnose acute tick-borne infections by paired serology and PCR (multiplex DNA PCR [mPCR] or reverse-transcription RNA PCR [RT-PCR]). Our primary analysis included those with optimal (13–42 days) convalescent sampling; others were included in a secondary analysis. We tested for *Babesia microti*, *Borrelia burgdorferi*, *Borrelia mayonii*, *Borrelia miyamotoi*, *Candidatus* Anaplasma capra, *Ehrlichia chaffeensis* (*EC*)*/Ehrlichia muris* subsp *eauclairensis* (*EME*), Heartland virus, Powassan virus, and spotted fever group rickettsioses (SFGR). Testing positive control sample aliquots stored at −20°C for similar durations regularly confirmed continued seroreactivity.

### *Borrelia* spp and *Babesia microti* Enzyme-Linked Immunosorbent Assay

We used enzyme-linked immunosorbent assay (ELISA) to test for *Borrelia* spp (C6 peptide ELISA) and *B microti* (Immunetics, Boston, Massachusetts). Based on consideration of a 4-fold antibody rise as “significant” and that optical density (OD) can estimate antibody quantity, we applied a statistical approach to define a significant rise in OD by ELISA [3]. Those specimens above a plate-to-plate normalized OD cutoff were considered positive (Lyme index value [C6IV] >0.9 or *B microti* signal-to-noise cutoff ratio [SCR] ≥1.6 [4]), as per the manufacturer. Paired sera in which the convalescent serum was positive and the convalescent minus acute-phase OD was greater than mean +3 standard deviations of the OD change in paired-negative samples were considered to have a significant C6IV or SCR increase akin to a 4-fold rise in titer [3].

### *B burgdorferi* Western Blot

We used a 2-tier test to confirm acute/recent *B burgdorferi* infection. Since C6 peptide ELISAs cannot distinguish *B burgdorferi* from *B miyamotoi* or *B mayonii* [5, 6], we tested available convalescent-phase sera by *B burgdorferi* IgG/immunoglobulin M (IgM) Western blot (WB) (MarDx/Trinity Biotech, Wicklow, Ireland) if either serum was C6 peptide ELISA positive.

### Indirect Fluorescent Antibody for *B mayonii*

Sera with significant C6IV antibody rises were examined for acute/recent *B mayonii* infection if a *B burgdorferi* WB was negative or not done. *Borrelia mayonii* was cultivated in BSK-II medium to supply antigen for IgG-IFA. Positive controls included immune mouse serum and 2 human anti–*B. mayonii* immune sera (courtesy Martin Schriefer and Jeannine Petersen, CDC, Fort Collins, Colorado). *Borrelia burgdorferi* cross-reactivity was assessed using sera from patients with culture- and serologically confirmed Lyme disease. Specificity tests used sera from uninfected mice; *B miyamotoi*, SFGR, *EC*, and *A phagocytophilum* infection; and healthy subjects. Sera were tested by *B mayonii* IFA at a 1:80 dilution; those with acute-phase titers ≤80 and convalescent-phase titers ≥80 were considered seroconversions.

### IFA for *Ehrlichia* spp

We tested for *EC* and *EME* by IgG IFA [7] (cultivating *EC* and *EME* in DH82 and RF/6A cells, respectively) [8]. Sera reactive at the 1:80 dilution were titrated to ≥2560. Those with 4-fold IgG antibody rises to ≥160 for *EC* and 10 *EC* IgG-negative controls were tested for *EME*. A ≥2-fold higher convalescent titer distinguished acute *EME* from *EC*.

### IFA for SFGR

Paired sera were screened at 1:80 by IgG IFA using acetone-fixed *Rickettsia parkeri* (Portsmouth strain)*-*infected human brain microvascular endothelial cells on multiwell glass slides. Reactive samples were titrated to 2560.

### mPCR to Detect Tick-borne Pathogens

We adapted our mPCR assay including SFGR (*sca0*) and *EC* [9] to include *B microti* (18S rRNA gene) [10], *B miyamotoi*, *B mayonii*, and *Candidatus* A capra (*gltA*). The *EC trp32* (*vlpt*) PCR also detected *EME*; thus, results were categorized as *EC*/*EME*. We used *glpQ* PCR for *B miyamotoi* with primers and probes common among *B miyamotoi* strains, but not other relapsing fever *Borrelia*, and adapted *oppA* PCR for *B mayonii* [11] by comparing *oppA* sequences for *B burgdorferi* sensu stricto and sensu lato strains. *Candidatus* A capra *gltA* PCR was adapted [12] to mPCR. AlleleID, IDT PrimerQuest, or NCBI Primer-BLAST were used to design assays that uniquely identified *B microti*, *B miyamotoi*, *B mayonii*, and *Candidatus* A capra. For RNA viruses, we targeted the RNA-dependent RNA polymerase gene of Powassan/deer tick virus (POWV; isolate DTV-MN-2008; HM991145.1) and the small segment nonstructural protein gene of the *Bandavirus* Heartland virus (HRTV). All primer/probe combinations were optimized for default parameters so that multiplexing had minimal impact on analytical sensitivity and retained high analytical specificity (Supplementary Table 1).

The QIAsymphony Midi DNA blood kit was used to extract DNA from 1 mL of EDTA-anticoagulated blood (final volume 200 µL). RNA was extracted from 200 µL archived acute-phase serum using the ZR-96 Viral RNA kit (Zymo Research).

We used a Bio-Rad CFX 384 Multicolor Real Time PCR instrument (default parameters, 40 cycles) for mPCR with duplicate or triplicate reactions. Single-step reverse-transcription mPCR (RT-PCR) was performed in quadruplicate; 2 wells received reverse transcriptase and 2 did not. All reactions included known positive, negative, and no-template controls and cloned amplicon quantitation standards to assure analytical sensitivity (≤10 copies/µL), linearity (*R*^2^ > 0.95), and efficiency (between 0.70 and 1.20). We used human *ACTB* as an amplification control in mPCR and RT-PCR assays; *ACTB*-negatives were repeated once. Positives were required to be in duplicate. No reproducible false positives were demonstrated among specificity controls (Supplementary Table 1).

### Reference Case Definitions

Acute/recent infections were confirmed by paired serology and/or PCR. Acute/recent *B burgdorferi* infection required (1) a positive convalescent C6IV result with a significant C6IV antibody rise or a high stable C6IV (both acute and convalescent C6IV in the top 50th percentile) and (2) a positive convalescent-phase *B burgdorferi* IgG and/or IgM WB. Acute/recent *B mayonii* infection required a significant C6IV antibody rise, a negative convalescent *B burgdorferi* IgG/IgM WB, and *B mayonii* IgG IFA seroconversion. We defined other acute/recent tick-borne infections as a significant SCR antibody increase (*B microti*) [13]; 4-fold IgG IFA rise (*EC/EME* or SFGR); a positive *B miyamotoi*, *EC*/*EME*, SFGR, *Candidatus* A capra, or *B microti* mPCR; or a positive POWV or HRTV RT-PCR. Seropositives without acute/recent infection were classified as past tick-borne infections. For *Borrelia*, those unconfirmed by WB were classified as possible acute/recent or past *Borrelia* based on the presence or absence, respectively, of a significant C6IV antibody rise.

## RESULTS

### Patients and Samples (Table 1)

Our primary analysis included specimens from 568 patients (184 in UM and 384 in NE) enrolled from May 1991 through July 2009, including 266 paired sera (median acute-convalescent sampling interval, 28 [interquartile range {IQR}, 21–33] days); duration of illness unknown). Among 85 UM patients with available data, the median age was 45 (IQR, 36–59) years. Consistent with suspected HGA, among 61 with complete blood counts, 15% had anemia, 54% leukopenia, and 61% thrombocytopenia. Both UM and NE patients presented almost exclusively (94%) in May–October. Specimens included paired sera with matched acute-phase blood or DNA (n = 41), paired sera alone (n = 225), acute-phase blood or DNA alone (n = 302), and acute-phase sera for POWV or HRTV RT- PCR (n = 233).

**Table 1.** Clinical Samples (N = 568) From Patients Tested for Tick-borne Infections After Excluding Anaplasmosis

Region	PCR^a^ and RT-PCR^b^		Serology^c^		
	Acute-Phase Blood or Blood DNA	Acute-Phase Serum for Viral RT-PCR	Paired Sera	Convalescent Serum (Median Days After Acute)	IQR, Days (min–max)
Total	343	233	266	28	21–33 (13–42)
Upper Midwest	8	183	179	29	23–34 (15–42)
Northeast	335	50	87	22	21–28 (13–42)

Abbreviations: IQR, interquartile range; PCR, polymerase chain reaction; RT-PCR, reverse-transcription polymerase chain reaction.

^a^DNA PCR: *Ehrlichia chaffeensis trp32*, *Ehrlichia muris* subsp *eauclairensis groE*, spotted fever group *Rickettsia sca0*, *Borrelia miyamotoi glpQ*, *Babesia microti* 18S rRNA gene, *ACTB* (human β-actin gene DNA control), *Borrelia mayonii oppA* (n = 211), *Candidatus* Anaplasma capra *gltA* (n = 211).

^b^Acute-phase serum for viral RNA and RT-PCR: Powassan/deer tick virus NS5 or 3′UTR; Heartland *Bandavirus* L, M, and S genomic segments; *ACTB* (human β-actin mRNA control).

^c^Paired serology: C6 peptide enzyme immunoassay (EIA), *B microti* EIA, *Borrelia burgdorferi* immunoglobulin G and immunoglobulin M Western blot, *E chaffeensis*, *E muris* subsp *eauclairensis*, spotted fever group *Rickettsia*, and *B mayonii* indirect fluorescent antibody.

### Summary of Acute Tick-borne Infections Identified

In total, 38 of 568 (7%) patients with suspected anaplasmosis in whom that etiology was ruled out had ≥1 agent of acute tick-borne illness identified, including 33 patients (35 infections) diagnosed by paired serology and 5 patients (7 infections) by PCR. These 42 acute/active infections included 24 *B burgdorferi*, 4 *B microti*, 5 *B miyamotoi*, 6 *EC/EME*, and 3 SFGR (Table 2). Four (10%) had paired serologic or molecular evidence of concurrent or closely sequential infections with distinct tick-borne pathogens.

**Table 2.** Results of Testing 568 Patients by Paired Serology (Convalescent Sample ≤42 Days; n = 266 Paired Sera) and by Multiplex Polymerase Chain Reaction (PCR) (n = 343 Acute-Phase Blood DNA) and Reverse-Transcription PCR (n = 233 Acute-Phase Serum RNA for Viral Reverse-Transcription PCR) for Tick-borne Infections That Mimic Anaplasmosis

Tick-borne Agent	PCR	RT-PCR^a^	Serology^b^	Paired Serology	Total
*Borrelia* C6 peptide positive^b^	ND	ND	100/266 (38%)	ND	100/266 (38%)
Significant *Borrelia* C6IV increase^c^	ND	ND	ND	60/266 (23%)	60/266 (23%)
*Borrelia burgdorferi* ^d^	ND	ND	ND	24/230 (10%)	24/230 (10%)
*Borrelia miyamotoi*	5/334 (1%)	ND	ND	ND	5/334 (1%)
*Borrelia mayonii*	0/173 (0%)	ND	ND	ND	0/173 (0%)
*Babesia microti*	2/334 (1%)	ND	ND	2/266 (<1%)	4/568 (1%)
*Ehrlichia chaffeensis/Ehrlichia muris* subsp *eauclairensis*	0/341 (0%)	ND	ND	6/266 (2%)	6/568 (1%)
Spotted fever *Rickettsia*	0/341 (0%)	ND	ND	3/266 (1%)	3/568 (<1%)
*Candidatus* Anaplasma capra	0/321 (0%)	ND	ND	ND	0/321 (0%)
POWV	ND	0/233 (0%)	ND	ND	0/233 (0%)
HRTV	ND	0/233 (0%)	ND	ND	0/233 (0%)

Data are presented as positives/No. tested (%).

Abbreviations: C6IV, C6 index value; HRTV, Heartland virus; ND, not done; PCR, polymerase chain reaction; POWV, Powassan/deer tick virus; RT-PCR, reverse-transcription polymerase chain reaction.

^a^RT-PCR using RNA prepared from acute-phase serum.

^b^Includes C6 positive tests in either acute or convalescent serum sample.

^c^Includes only paired sera with a significant increase in C6IV from acute to convalescent sample.

^d^Includes 7 patients with positive acute and convalescent C6 peptide enzyme-linked immunosorbent assay (ELISA) results consistent with stable high titer that were confirmed by *Borrelia burgdorferi* Western blot; n = 230 because Western blot could not be performed in 7 C6 peptide ELISA-positives with significant increase in C6IV and 29 without significant increase in C6IV antibodies.

### Acute/Recent Infections by Paired Serology

**Figure 1.**
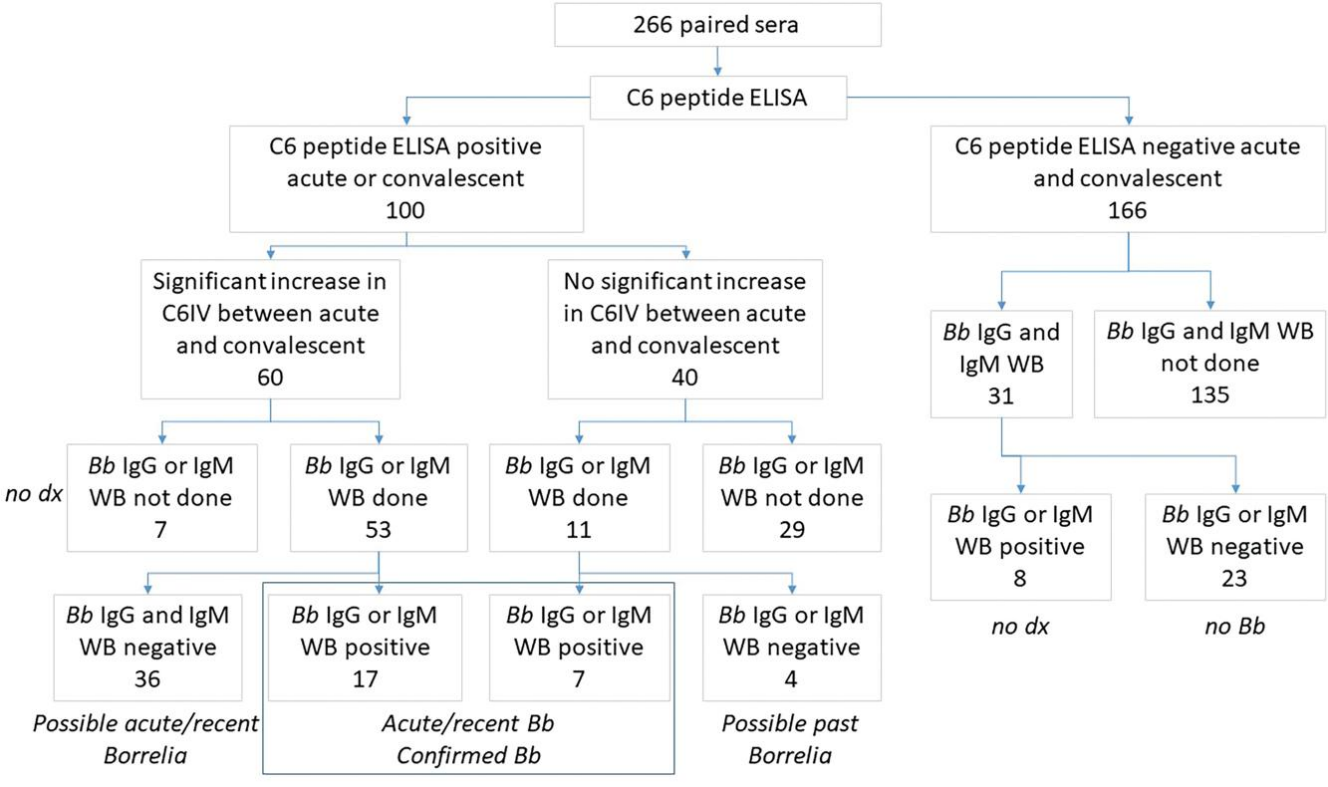
Sample numbers and schematic describing testing approach for *Borrelia burgdorferi* serology using the C6 peptide enzyme-linked immunosorbent assay and *B burgdorferi* Western blot. Abbreviations: *Bb*, *Borrelia burgdorferi*; C6IV, Lyme index value for the C6 peptide enzyme-linked immunosorbent assay; dx, diagnosis; ELISA, enzyme-linked immunosorbent assay; IgG, immunoglobulin G: IgM, immunoglobulin M; WB, Western blot.

**Figure 2.**
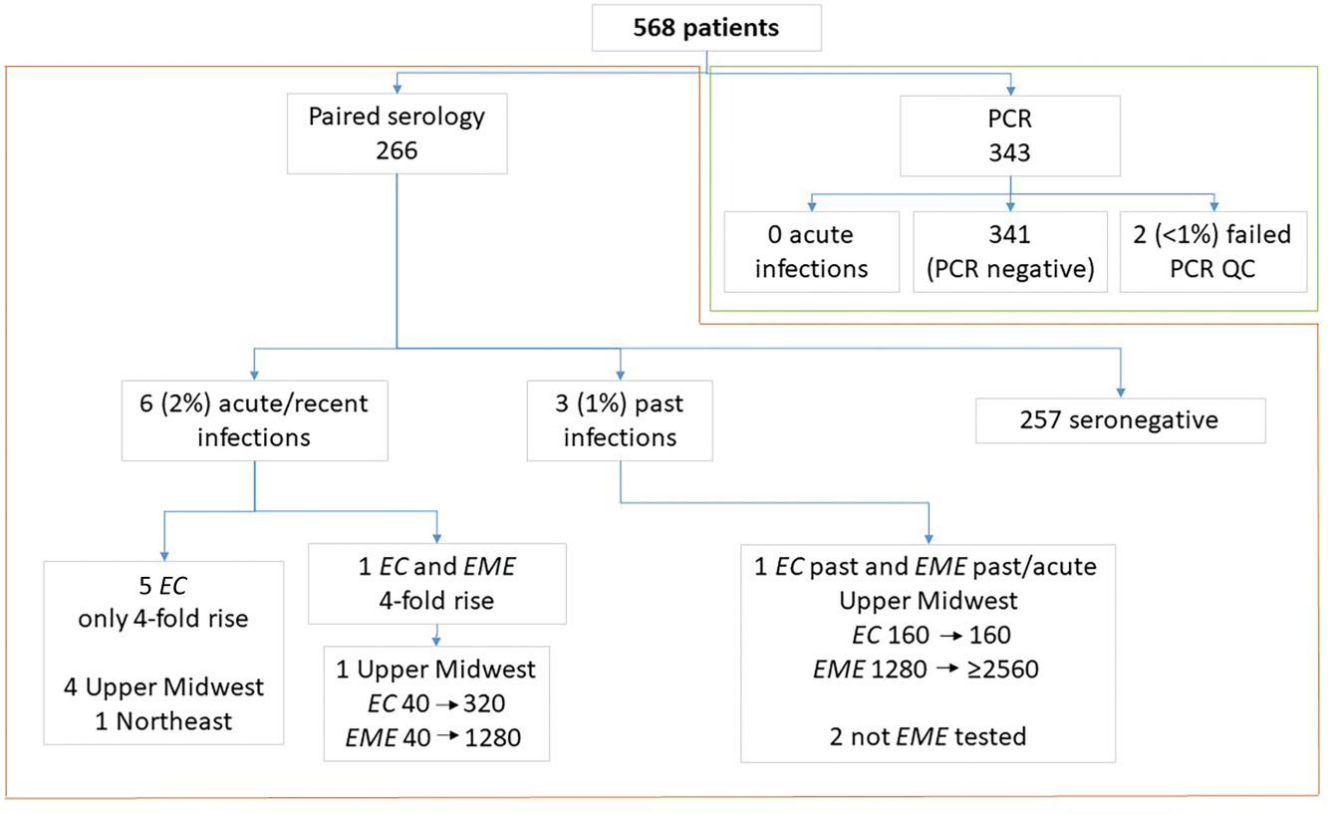
Sample numbers and schematic describing testing approach for *Ehrlichia chaffeensis* and *Ehrlichia muris* subsp *eauclairensis* using polymerase chain reaction testing and paired (acute and convalescent) serology. Abbreviations: *EC*, *Ehrlichia chaffeensis*; *EME*, *Ehrlichia muris* subsp *eauclairensis*; PCR, polymerase chain reaction; QC, quality control.

Of C6 peptide ELISA-positives, 24 of 230 (10%) had WB-confirmed acute/recent *B burgdorferi* infections, including 17 of 24 (71%) with significant C6IV antibody rises and 7 stable high titers; 36 of 266 lacked WB. Paired serology also identified 11 other acute/recent infections, including 2 of 266 (>1%) *B microti*, 6 of 266 (2%) *EC*/*EME* (5 *EC* and 1 *EME*), and 3 of 266 (1%) SFGR (Table 2, Figures 1 and 2).

### Acute Infections by mPCR

Using mPCR, we identified 7 acute infections (5/334 [1%] *B miyamotoi* and 2/334 [1%] *B miyamotoi*/*B microti*) in 5 patients presenting during 1995–1997; for all other pathogens, mPCR or RT-PCR was negative (*EC* [0/341], SFGR [0/341], *Candidatus* A capra [0/321], *B mayonii* [0/173], and POWV and HRTV [0/233 each]) (Table 2).

### Correlation of Paired Serology and mPCR

Of 41 patients tested by both paired serology and mPCR, 4 (10%) had evidence of acute/recent infection; none were confirmed by both serology and PCR. Acute/recent infection was confirmed by paired serology in 3 patients (1 *B burgdorferi*, 1 *EC/EME*, and 1 SFGR) and by mPCR in 1 (*B miyamotoi* and *B microti*).

### Acute/Recent Single and Potential Coinfections by Paired Serology and mPCR

Of 38 patients with acute/early infection, single infections were identified in 34 (22 *B burgdorferi*, 5 *EC/EME*, 2 *B microti*, 3 *B miyamotoi*, and 2 SFGR). Four had evidence of concurrent or sequential infection with 2 pathogens, including 2 with *B burgdorferi* (1 *EC*/*EME* [UM] and 1 SFGR [UM]) by paired serology, with 23- and 24 day-convalescent sera, respectively, and 2 with *B microti* and *B miyamotoi* (both NE) by PCR.

In total, 38 of 568 (7%) patients with suspected anaplasmosis in whom that etiology was excluded had ≥1 agent of tick-borne illness, with 33 patients (35 infections) identified by paired serology and 5 patients (7 infections) by PCR. These 42 acute/active infections included 24 *B burgdorferi*, 4 *B microti*, 5 *B miyamotoi*, 6 *EC/EME*, and 3 SFGR (Table 2). Four (10%) had serologic or molecular evidence of infections by distinct tick-borne pathogens concurrently or in close sequence.

### Geographic Differences

Of 184 patients in the UM, 18 (10%) had tick-borne infections versus 20 of 384 (5%) patients in the NE, excluding those tested exclusively by RT-PCR. These included 11 *B burgdorferi* infections in the UM (6%) and 13 (3%) in the NE (*P* = .150; Table 3).

**Table 3.** Agents of Tick-borne Disease Identified by Paired Serology and/or Polymerase Chain Reaction From Patients With Suspected Anaplasmosis

Characteristic	Total, No.	Upper Midwest, No.	Northeast, No.
Total patients	38	18	20
Total infections	42	20	22
Patients with a single infection	34	16	18
* Borrelia burgdorferi*	22	9	13
* Borrelia miyamotoi*	3	0	3
* Babesia microti*	2	2	0
* Ehrlichia chaffeensis/Ehrlichia muris* subsp *eauclairensis*	5	4	1
* *Spotted fever *Rickettsia*	2	1	1
Patients with coinfections	4	2	2
* B burgdorferi* and *E chaffeensis/E muris* subsp *eauclairensis*	1	1	0
* B burgdorferi* and spotted fever *Rickettsia*	1	1	0
* B microti* and *B miyamotoi*	2	0	2

### Past Infections

There were 3 (1%) past *B microti* infections. The 3 (1%) *EC*/*EME* past infections, all in patients from the UM, included 1 with a stable EC titer (160) who had a ≥2-fold *EME* titer (1280 to ≥2560) increase (Figure 2). Past SFGR infections were identified in 2 of 266 (1%) patients.

### Other *Borrelia* Infections

Forty-three (16.2%) patients had possible acute/recent *Borrelia* and 33 (12.4%) past *Borrelia* infection (Figure 1).

### Secondary Analysis

Among 645 patients (357 with paired sera <180 days; median acute-convalescent sampling interval, 31 [IQR, 23–43; max, 175] days), 51 (7.9%) had ≥1 tick-borne agent identified. Paired serology–identified acute/recent infections included 30 (10%) *B burgdorferi*, 3 (1%) *B microti*, 7 (2%) *EC/EME*, and 4 (1%) SFGR (Supplementary Tables 2 and 3).

## DISCUSSION

Tick-transmitted infections in the US are increasingly recognized [14, 15]. Molecular tools have revolutionized taxonomy, identified new pathogens, and improved epidemiologic understanding of pathogens. Investigation of atypical presentations of SFGR or typical presentations in new regions led to the identification of *E chaffeensis* in 1986, *A phagocytophilum* in 1990, *Ehrlichia ewingii* in 1999, *EME* in 2009, and *Candidatus* A capra in China in 2015 [7, 12, 16]. Lyme disease, caused by *B burgdorferi* sensu stricto, is the most frequently reported tick-borne disease in the US; 2 other *Borrelia* species, *B miyamotoi* and *B mayonii*, are now reported as causes of relapsing fever– or Lyme disease–like illnesses in the US, respectively [5, 17]. *Babesia microti* cases increased 4- to 20-fold in the last decade [14]. Tick-borne viruses, including Powassan and deer tick viruses, can cause fever and severe neurologic illness in the NE and UM [18]. In 2012, a new tick-borne virus, “Heartland” *Bandavirus*, was identified in >60 patients in the US [19]; the tick vector and disease distribution in the US remains understudied [20]. Recent identification of new *Rickettsia* spp, with known and unknown pathogenicity, complicates testing, reporting, and epidemiologic study [21, 22].

We used state-of-the-art methods (paired serology and PCR) to test patients living in tick-borne disease–endemic areas with suspected anaplasmosis for other tick-borne agents after excluding anaplasmosis. Importantly, this approach can (1) identify previously undetected pathogens (eg, *Candidatus* A capra), (2) better define the date of pathogen introduction in an area (eg, *B miyamotoi*), and/or (3) determine the relative frequency of tick-borne infections in a specific population.

An alternative tick-borne infection (*B burgdorferi*, *EC/EME*, *B microti*, *B miyamotoi*, and SFGR) was identified in 38 of 568 patients on primary analysis, and 51 on secondary analysis (Supplementary Tables 2 and 3). This is an important proof-of-concept that if extensive testing is performed in patients at high risk for tick-borne infection with a compatible clinical syndrome, a definitive diagnosis can be achieved in a small but nontrivial percentage. Furthermore, it provides additional data that between 1991 and 2009, tick-borne diseases were underdiagnosed in a high-prevalence setting. The substantial number of patients with *Borrelia* C6 peptide antibodies in patients for whom WB was not positive could represent incomplete evolution of *B burgdorferi* antibody response, *B miyamotoi* or *B mayonii* infections, unknown infections, or, less likely, false positives [5, 17]. We omitted *B burgdorferi* PCR, since it is generally insensitive due to transient low-level bacteremia [23]. In contrast, we applied *B mayonii* PCR since that pathogen often achieves higher-level bacteremia; however, we did not detect any confirmed infections by PCR or serology [5].

We identified evidence of acute/recent, simultaneous or closely sequential infections in 4 (10%) patients; 2 were PCR positive for both *B microti* and *B miyamotoi* and 2 had paired serology consistent with rapid succession or simultaneous *B burgdorferi* and *EC*/*EME* (1) or SFGR (1) infection. Coinfections with *Ixodes* tick-transmitted agents (eg, *B burgdorferi*, *B miyamotoi*, *B mayonii*, *B microti*, and *EME*) are biologically plausible and documented [24], but presumed to be infrequent [25]. A *Borrelia* C6 peptide–positive result in a *B miyamotoi* PCR-positive patient could reflect *B miyamotoi* alone, since *Borrelia* spp, like SFGR, stimulate cross-reactive antibodies [17]. The coinfection prevalence of 10% (95% confidence interval, 4%–22%) is consistent with other studies [25, 26]. Since patients with anaplasmosis were excluded from the study, we likely underestimate the number with coinfection.

To decrease misclassification of sequential infections as coinfections and past infections as acute, we used reference standard definitions, including a 4-fold rise in IgG antibody titer by IFA and/or positive mPCR, to confirm acute SFGR and *Ehrlichia* infections, and our primary analysis included those with optimal (≤42 days) convalescent sampling. The problem of resolving coinfections versus rapidly sequential infections is illustrated by “coinfections” of *B burgdorferi*, transmitted by *I scapularis*, with *EC* and SFGR that are transmitted largely by *Amblyomma americanum* and *Dermacentor variabilis*, respectively, with overlapping distributions [27]. Although historically rare in both regions, SFGR are plausible as single infections, since the most common vector, *D variabilis*, is well-documented in both regions [28]. Although also plausible, SFGR are not known to be transmitted by *I scapularis* tick bites. As precedent, transmission of *Rickettsia australis* from *Ixodes holocyclus* is accepted but not proven [29], and human SFGR infection from other *Ixodes* spp, such as *Rickettsia monacensis* and *Rickettsia helvetica* from *Ixodes ricinus*, is controversial [30, 31]. Moreover, since PCR for SFGR is poorly sensitive [9], cases here were identified only by serology. The acute SFGR infections here occurred during a 2-decade national trend of increased reporting [32], which may reflect infections with less pathogenic species such as *R parkeri*, subclinical infections, immune stimulation triggered by nonpathogenic spotted fever group tick endosymbionts [22], or immune stimulation rather than increased incidence or reporting of *Rickettsia rickettsii* infection (Rocky Mountain spotted fever) in much of the US [33]. Since most reported SFGR cases are based on testing single sera [32] and many healthy individuals have detectable antibody [34], we rigorously tested paired sera to confirm acute SFGR infections.

Likewise, since *A americanum*, the *EC* vector, was infrequent in the UM when these samples were obtained, serologic reactivity in samples from the UM could reflect *EME* infection. Examples include 2 patients from the UM, 1 with an *EC* 4-fold titer increase from <80 to 320 concurrent with a ≥16-fold *EME* titer increase from <80 to 1280, and another with at least a 2-fold *EME* titer increase (1280 to ≥2560) but stable *EC* titers of 160. The lack of *EME* serologic responses in the NE is consistent with its apparent restriction to the UM. However, although *EC* infection is now established in the NE [35], *A americanum* and *EC* were rarely identified there during the interval of this study.

Using molecular tools, we sought evidence for 2 new *Borrelia* spp (*B mayonii*, only identified in the UM, and *B miyamotoi*), a new *Ehrlichia* spp (*EME*, only found in the UM), a novel *Candidatus* Anaplasma spp (identified in Asia and recently in Europe [12, 36]), and 2 tick-borne viruses emerging in the US [37]. We would expect *B miyamotoi* to cause anaplasmosis-like acute febrile illness in the UM and NE [38]; we identified cases in the NE US between 1995 and 1997, more than a decade prior to the first description of *B miyamotoi* in humans in Russia and subsequently in the US [38–40]. We found that *B miyamotoi* comprised 17% of the clearly identified *Borrelia* infections. Although PCR on blood for POWV and HRTV is rarely done for those without encephalitis, these infections could have antecedent or accompanying viremia. That we did not identify POWV or HRTV in our 1991–2009 cohorts suggest these viruses were not unrecognized causes of illness in our study population. Similarly, *Candidatus* A capra infections, a novel form of human anaplasmosis not previously identified as a cause of infections in humans in the US, was not found using PCR. Unavailability of control samples precluded determination of clinical sensitivity for new molecular assays; however, analytical sensitivity at ≤10 genomic copies/µL and clinical specificity >90%–95% appeared high.

Limitations for this study include the small number of individual diagnoses for several key infections identified in this 1991–2009 cohort, the lack of consensus for considering some serologic tests positive [41], the inability to validate the statistically plausible ELISA-based estimation of 4-fold antibody rise for the C6 peptide and *B microti* assays, the unknown duration of illness before acute-phase sampling, the lack of PCR testing for *B burgdorferi* and insufficient sera to test all by *B burgdorferi* WB, the lack of specific PCR and serologic assays to differentiate *EC* from *EME*, difficulties diagnosing specific SFGRs even with gold-standard paired IFA, and the unavailability of robust *B miyamotoi* and *B mayonii* serological tests to discriminate them from *B burgdorferi* (which might be overcome by using antigens more unique to *B miyamotoi*). Other infectious agents that emerged since 2009 include *Anaplasma bovis* in humans, which might have been detected if an *Anaplasma* genus-level nucleic acid amplification test were used. Similarly, if more recent samples had been available, it is possible that the pathogens sought might have been detected more often. Importantly, this study highlights the imperfect state-of-the-art regarding diagnosis of tick-borne infections: (1) serology is intrinsically nonspecific, since human immune responses vary greatly and cannot differentiate some closely related organisms, and (2) PCR-based tools, though highly sensitive and specific for detecting some pathogens (eg, *A phagocytophilum*, *B microti*, and *B miyamotoi*) early after illness onset, may be less sensitive later in illness. Furthermore, PCR assays for other pathogens, especially for *B burgdorferi* sensu stricto and SFGR, remain insensitive due to low-level bacteremia. Currently, newer technologies that employ relatively unbiased approaches for identifying unsuspected pathogens, such as metagenomics, need further optimization and validation [42]. However, such approaches offer promise for identifying additional novel tick-borne infections that mimic anaplasmosis.

In conclusion, after excluding those with anaplasmosis, we used reference standard testing (paired serology and/or PCR) to identify other tick-borne infections in 38 of 568 patients. Although most diagnoses were accomplished with paired serology, our results suggest that PCR and paired serology are complementary for the detection of acute/recent tick-borne infections. Since the epidemiologic landscape of tick-borne infections in the US has changed considerably recently, these data may not be generalizable to 2023–2024. However, the findings are highly relevant, since the spectrum of tick-borne pathogens has only increased, and limitations in state-of-the-art testing approaches remain major impediments to accurate diagnosis. Thus, epidemiologic and clinical studies using improved and state-of-the-art assays will be required to define the breadth and relative frequency of emerging tick-borne agents in the US as well as the identification and ecology of relevant vectors. Prospective studies to validate and extend these findings should be performed to improve clinical understanding and management of tick-borne infections, discern trends in tick-borne disease epidemiology, and to search for novel agents.

## Supplementary Material

ofae149_Supplementary_Data

## Supplementary Data

Supplementary materials are available at *Open Forum Infectious Diseases* online. Consisting of data provided by the authors to benefit the reader, the posted materials are not copyedited and are the sole responsibility of the authors, so questions or comments should be addressed to the corresponding author.

## Notes

***Acknowledgments.*** The authors thank Meg Lichay for excellent technical support and Gary P. Wormser for supportive and detailed discussion and advice about data interpretation.

***Patient consent.*** The institutional review boards of Johns Hopkins Medicine (Baltimore, Maryland), Duluth Clinic/St Mary's Hospital (Duluth, Minnesota), St Luke's Hospital (Duluth, Minnesota), New York Medical College (Valhalla, New York), and New York State Department of Health (Albany, New York) reviewed and either approved the research or declared the research exempt.

***Disclaimer.*** The opinions expressed herein are those of the author(s) and are not necessarily representative of those of the Uniformed Services University of the Health Sciences; the Department of Defense; or the US Army, Navy, or Air Force. The funders had no role in study design, data collection and interpretation, or the decision to submit the work for publication.

***Financial support*.** This work and testing were supported by a Fisher Center Discovery Program (https://hopkinsinfectiousdiseases.jhmi.edu/research/research-areas/environmental-id/fisher-center-discovery-programgrants/) award to M. E. R. Additional laboratory support was provided through the National Institute of Allergy and Infectious Diseases (grant numbers R01 AI044102 and R21 AI09606) to J. S. D.

## References

[1] Bakken JS , Dumler JS, Chen SM, Eckman MR, Van Etta LL, Walker DH. Human granulocytic ehrlichiosis in the upper Midwest United States. A new species emerging? JAMA 1994; 272:212–8. 8022040

[2] Bakken JS , Aguero-Rosenfeld ME, Tilden RL, et al Serial measurements of hematologic counts during the active phase of human granulocytic ehrlichiosis. Clin Infect Dis 2001; 32:862–70. 11247709 10.1086/319350

[3] Bloch EM , Levin AE, Williamson PC, et al A prospective evaluation of chronic *Babesia microti* infection in seroreactive blood donors. Transfusion 2016; 56:1875–82. 27184253 10.1111/trf.13617 PMC6014595

[4] Levin AE , Williamson PC, Bloch EM, et al Serologic screening of United States blood donors for *Babesia microti* using an investigational enzyme immunoassay. Transfusion 2016; 56:1866–74. 27224258 10.1111/trf.13618 PMC6007971

[5] Pritt BS , Mead PS, Johnson DK, et al Identification of a novel pathogenic *Borrelia* species causing Lyme borreliosis with unusually high spirochaetaemia: a descriptive study. Lancet Infect Dis 2016; 16:556–64. 26856777 10.1016/S1473-3099(15)00464-8 PMC4975683

[6] Molloy PJ , Weeks KE, Todd B, Wormser GP. Seroreactivity to the C6 peptide in *Borrelia miyamotoi* infections occurring in the northeastern United States. Clin Infect Dis 2018; 66:1407–10. 29149281 10.1093/cid/cix1023

[7] Pritt BS , Sloan LM, Johnson DK, et al Emergence of a new pathogenic *Ehrlichia* species, Wisconsin and Minnesota, 2009. N Engl J Med 2011; 365:422–9. 21812671 10.1056/NEJMoa1010493 PMC3319926

[8] Wong SJ , Brady GS, Dumler JS. Serological responses to *Ehrlichia equi*, *Ehrlichia chaffeensis*, and *Borrelia burgdorferi* in patients from New York state. J Clin Microbiol 1997; 35:2198–205. 9276387 10.1128/jcm.35.9.2198-2205.1997 PMC229939

[9] Reller ME , Dumler JS. Optimization and evaluation of a multiplex quantitative PCR assay for detection of nucleic acids in human blood samples from patients with spotted fever rickettsiosis, typhus rickettsiosis, scrub typhus, monocytic ehrlichiosis, and granulocytic anaplasmosis. J Clin Microbiol 2020; 58:e01802-19. 32493778 10.1128/JCM.01802-19 PMC7448621

[10] Bloch EM , Lee TH, Krause PJ, et al Development of a real-time polymerase chain reaction assay for sensitive detection and quantitation of *Babesia microti* infection. Transfusion 2013; 53:2299–306. 23362840 10.1111/trf.12098

[11] Pritt BS , Respicio-Kingry LB, Sloan LM, et al *Borrelia mayonii* sp. nov., a member of the *Borrelia burgdorferi* sensu lato complex, detected in patients and ticks in the upper midwestern United States. Int J Syst Evol Microbiol 2016; 66:4878–80. 27558626 10.1099/ijsem.0.001445 PMC5214957

[12] Li H , Zheng YC, Ma L, et al Human infection with a novel tick-borne *Anaplasma* species in China: a surveillance study. Lancet Infect Dis 2015; 15:663–70. 25833289 10.1016/S1473-3099(15)70051-4

[13] Mead P , Petersen J, Hinckley A. Updated CDC recommendation for serologic diagnosis of Lyme disease. MMWR Morb Mortal Wkly Rep 2019; 68:703. 31415492 10.15585/mmwr.mm6832a4 PMC6818702

[14] Rosenberg R , Lindsey NP, Fischer M, et al Vital signs: trends in reported vectorborne disease cases—United States and territories, 2004–2016. MMWR Morb Mortal Wkly Rep 2018; 67:496–501. 29723166 10.15585/mmwr.mm6717e1 PMC5933869

[15] Kugeler KJ , Schwartz AM, Delorey MJ, Mead PS, Hinckley AF. Estimating the frequency of Lyme disease diagnoses, United States, 2010–2018. Emerg Infect Dis 2021; 27:616–9. 33496229 10.3201/eid2702.202731 PMC7853543

[16] Dumler JS , Bakken JS. Human ehrlichioses: newly recognized infections transmitted by ticks. Annu Rev Med 1998; 49:201–13. 9509259 10.1146/annurev.med.49.1.201

[17] Krause PJ , Narasimhan S, Wormser GP, et al *Borrelia miyamotoi* sensu lato seroreactivity and seroprevalence in the northeastern United States. Emerg Infect Dis 2014; 20:1183–90. 24960072 10.3201/eid2007.131587 PMC4073859

[18] Piantadosi A , Rubin DB, McQuillen DP, et al Emerging cases of Powassan virus encephalitis in New England: clinical presentation, imaging, and review of the literature. Clin Infect Dis 2016; 62:707–13. 26668338 10.1093/cid/civ1005 PMC4850925

[19] Centers for Disease Control and Prevention. Heartland virus disease (Heartland) statistics and maps. Available at: https://www.cdc.gov/heartland-virus/statistics/index.html. Accessed 16 February 2023.

[20] Pastula DM , Turabelidze G, Yates KF, et al Notes from the field: Heartland virus disease—United States, 2012–2013. MMWR Morb Mortal Wkly Rep 2014; 63:270–1. 24670929 PMC5779346

[21] Verhoeve VI , Fauntleroy TD, Risteen RG, Driscoll TP, Gillespie JJ. Cryptic genes for interbacterial antagonism distinguish *Rickettsia* species infecting blacklegged ticks from other *Rickettsia* pathogens. Front Cell Infect Microbiol 2022; 12:880813. 35592653 10.3389/fcimb.2022.880813 PMC9111745

[22] Apperson CS , Engber B, Nicholson WL, et al Tick-borne diseases in North Carolina: is “*Rickettsia amblyommii*” a possible cause of rickettsiosis reported as Rocky Mountain spotted fever? Vector Borne Zoonotic Dis 2008; 8:597–606. 18447622 10.1089/vbz.2007.0271

[23] Liveris D , Schwartz I, McKenna D, et al Comparison of five diagnostic modalities for direct detection of *Borrelia burgdorferi* in patients with early Lyme disease. Diagn Microbiol Infect Dis 2012; 73:243–5. 22571973 10.1016/j.diagmicrobio.2012.03.026 PMC3377843

[24] Diuk-Wasser MA , Vannier E, Krause PJ. Coinfection by *Ixodes* tick-borne pathogens: ecological, epidemiological, and clinical consequences. Trends Parasitol 2016; 32:30–42. 26613664 10.1016/j.pt.2015.09.008 PMC4713283

[25] Wormser G , McKenna D, Scavarda C, et al Co-infections in persons with early Lyme disease, New York, USA. Emerg Infect Dis 2019; 25:748–52. 30882316 10.3201/eid2504.181509 PMC6433014

[26] Goldstein EJC , Thompson C, Spielman A, Krause PJ. Coinfecting deer-associated zoonoses: Lyme disease, babesiosis, and ehrlichiosis. Clin Infect Dis 2001; 33:676–85. 11486290 10.1086/322681

[27] Ogden NH , Mechai S, Margos G. Changing geographic ranges of ticks and tick-borne pathogens: drivers, mechanisms and consequences for pathogen diversity. Front Cell Infect Microbiol 2013; 3:46. 24010124 10.3389/fcimb.2013.00046 PMC3756306

[28] Drexler NA , Dahlgren FS, Heitman KN, Massung RF, Paddock CD, Behravesh CB. National surveillance of spotted fever group rickettsioses in the United States, 2008–2012. Am J Trop Med Hyg 2016; 94:26–34. 26324732 10.4269/ajtmh.15-0472 PMC4710440

[29] Stewart A , Armstrong M, Graves S, Hajkowicz K. *Rickettsia australis* and Queensland tick typhus: a rickettsial spotted fever group infection in Australia. Am J Trop Med Hyg 2017; 97:24–9. 28719297 10.4269/ajtmh.16-0915 PMC5508907

[30] Markowicz M , Schötta AM, Höss D, et al Infections with tickborne pathogens after tick bite, Austria, 2015–2018. Emerg Infect Dis 2021; 27:1048–56. 33755546 10.3201/eid2704.203366 PMC8007293

[31] Koetsveld J , Tijsse-Klasen E, Herremans T, Hovius JW, Sprong H. Serological and molecular evidence for spotted fever group *Rickettsia* and *Borrelia burgdorferi* sensu lato co-infections in the Netherlands. Ticks Tick Borne Dis 2016; 7:371–7. 26739030 10.1016/j.ttbdis.2015.12.010

[32] Binder AM , Heitman KN, Drexler NA. Diagnostic methods used to classify confirmed and probable cases of spotted fever rickettsioses—United States, 2010–2015. MMWR Morb Mortal Wkly Rep 2019; 68:243–6. 30870409 10.15585/mmwr.mm6810a3 PMC6421962

[33] Openshaw JJ , Swerdlow DL, Krebs JW, et al Rocky Mountain spotted fever in the United States, 2000–2007: interpreting contemporary increases in incidence. Am J Trop Med Hyg 2010; 83:174–82. 20595498 10.4269/ajtmh.2010.09-0752 PMC2912596

[34] Straily A , Stuck S, Singleton J, et al Antibody titers reactive with *Rickettsia rickettsii* in blood donors and implications for surveillance of spotted fever rickettsiosis in the United States. J Infect Dis 2019; 221:1371–8. 10.1093/infdis/jiz316 31267128

[35] Aliota MT , Dupuis AP II, Wilczek MP, Peters RJ, Ostfeld RS, Kramer LD. The prevalence of zoonotic tick-borne pathogens in *Ixodes scapularis* collected in the Hudson Valley, New York state. Vector Borne Zoonotic Dis 2014; 14:245–50. 24689680 10.1089/vbz.2013.1475 PMC3993027

[36] Jouglin M , Blanc B, de la Cotte N, Bastian S, Ortiz K, Malandrin L. First detection and molecular identification of the zoonotic *Anaplasma capra* in deer in France. PLoS One 2019; 14:e0219184. 31276519 10.1371/journal.pone.0219184 PMC6611577

[37] Wormser GP , Pritt B. Update and commentary on four emerging tick-borne infections: *Ehrlichia muris*-like agent, *Borrelia miyamotoi*, deer tick virus, Heartland virus, and whether ticks play a role in transmission of *Bartonella henselae*. Infect Dis Clin North Am 2015; 29:371–81. 25999230 10.1016/j.idc.2015.02.009

[38] Chowdri HR , Gugliotta JL, Berardi VP, et al *Borrelia miyamotoi* infection presenting as human granulocytic anaplasmosis: a case report. Ann Intern Med 2013; 159:21–7. 23817701 10.7326/0003-4819-159-1-201307020-00005

[39] Gugliotta JL , Goethert HK, Berardi VP, Telford SR III. Meningoencephalitis from *Borrelia miyamotoi* in an immunocompromised patient. N Engl J Med 2013; 368:240–5. 23323900 10.1056/NEJMoa1209039 PMC4018741

[40] Platonov AE , Karan LS, Kolyasnikova NM, et al Humans infected with relapsing fever spirochete *Borrelia miyamotoi*, Russia. Emerg Infect Dis 2011; 17:1816–23. 22000350 10.3201/eid1710.101474 PMC3310649

[41] Aguero-Rosenfeld ME , Donnarumma L, Zentmaier L, et al Seroprevalence of antibodies that react with *Anaplasma phagocytophila*, the agent of human granulocytic ehrlichiosis, in different populations in Westchester County, New York. J Clin Microbiol 2002; 40:2612–5. 12089287 10.1128/JCM.40.7.2612-2615.2002 PMC120546

[42] Kingry L , Sheldon S, Oatman S, et al Targeted metagenomics for clinical detection and discovery of bacterial tick-borne pathogens. J Clin Microbiol 2020; 58:e00147-20. 32878950 10.1128/JCM.00147-20 PMC7587092

