# Supplementary material for: Emerging Tick-borne Infections in the Upper Midwest and Northeast United States Among Patients With Suspected Anaplasmosis
Source: Open Forum Infect Dis. 2024 Mar 15;11(4):ofae149. doi: 10.1093/ofid/ofae149 (PMC11034950; doi:10.1093/ofid/ofae149)
Supplement: ofae149_Supplementary_Data [file ofae149_supplementary_data.zip › Supplemental Table 2.docx]

| **Supplemental Table 2. Clinical samples (n=645) from patients suspected to have had anaplasmosis but in whom that etiology was ruled out** **who were tested for other tick-borne infections. Includes patients with convalescent serum obtained up to 180 days after illness onset.** | | | | | |
| --- | --- | --- | --- | --- | --- |
| **Region** | **PCR^1^ and rt-PCR^2^** | | **Serology^3^** | | |
|  | **Acute phase blood or blood DNA** | **Acute phase serum for viral rt-PCR** | **Paired sera** | **Convalescent serum (median days after acute)** | **IQR** |
|  |  |  |  |  | **(min-max) days** |
| Total | 343 | 233 | 357 | 31 | 23-43 (13-175) |
| Upper Midwest | 8 | 183 | 242 | 33 | 26-44 (15-175) |
| Northeast | 335 | 50 | 115 | 27 | 21-41 (13-146) |
| ^1^ DNA PCR –*E. chaffeensis trp32*, *E. muris* *eauclairensis* *groE*, spotted fever group *Rickettsia sac0*, *B. microti* 18S rRNA gene, *ACTB* (human beta actin gene DNA control) | | | | | |
| ^2^ acute phase serum for viral RNA and rt-PCR (reverse transcriptase PCR) – Powassan/deer tick virus NS5 or 3’UTR; Heartland *Bandavirus* L, M, and S genomic segments; *ACTB* (human beta actin mRNA control) | | | | | |
| ^3^ paired serology – C6 peptide EIA, *B. microti* EIA, *A. phagocytophilum*, *E. chaffeensis, E. muris* subsp. *eauclairensis* and spotted fever group *Rickettsia* IFA | | | | | |
