# Supplementary material for: Emerging Tick-borne Infections in the Upper Midwest and Northeast United States Among Patients With Suspected Anaplasmosis
Source: Open Forum Infect Dis. 2024 Mar 15;11(4):ofae149. doi: 10.1093/ofid/ofae149 (PMC11034950; doi:10.1093/ofid/ofae149)
Supplement: ofae149_Supplementary_Data [file ofae149_supplementary_data.zip › Supplemental Table 3.docx]

| **Supplemental Table 3. Results of testing n=645 patients by: 1) paired serology (convalescent sample ≤ 180 days; n=357 paired sera), 2) by multiplex PCR (n=343 acute phase blood DNA) and reverse transcriptase PCR (n=233 acute phase serum RNA for viral rt-PCR) for tick-borne pathogens (+/no. tested (%)). Gray boxes = not done.** | | | | | | |
| --- | --- | --- | --- | --- | --- | --- |
| **Tick-borne agent** | | **PCR** | **rt-PCR^4^** | **Serology^1^** | **Paired serology** | **TOTAL** |
| *Borrelia* C6 peptide positive ^1^ | |  |  | 123/357 (34%) |  | 123/357 (34%) |
|  | significant C6IV increase^2^ |  |  |  | 74/357 (21%) | 74/357 (21%) |
| *Borrelia burgdorferi*^3^ | |  |  |  | 30/311 (10%) | 30/311 (10%) |
| *Borrelia miyamotoi* | | 5/334 (2%) |  |  |  | 5/334 (2%) |
| *Borrelia mayonii* | | 0/173 (0%) |  |  |  | 0/173 (0%) |
| *Babesia microti* | | 2/341 (1%) |  |  | 3/357 (1%) | 5/645 (1%) |
| *E. chaffeensis / E. muris* subsp*. eauclairensis* | | 0/341 (0%) |  |  | 7/357 (2%) | 7/645 (1%) |
| Spotted fever *Rickettsia* | | 0/341 (0%) |  |  | 4/357 (1%) | 4/645 (1%) |
| *Candidatus A. capra* | | 0/321 (0%) |  |  |  | 0/321 (0%) |
| POWV | |  | 0/233 (0%) |  |  | 0/233 (0%) |
| HRTV | |  | 0/233 (0%) |  |  | 0/233 (0%) |
| \| ^1^ DNA PCR –*E. chaffeensis trp32*, *E. muris* ssp. *eauclairensis* *groE*, spotted fever group *Rickettsia sca0*, *B. miyamotoi glpQ, B. microti* 18S rRNA gene, *ACTB* (human beta actin gene DNA control), *B. mayonii oppA* (n=211), *Candidatus* A. capra *gltA* (n=211) \| \| --- \| \| ^2^ acute phase serum for viral RNA and rt-PCR (reverse transcriptase PCR) – Powassan/deer tick virus NS5 or 3’UTR; Heartland *Bandavirus* L, M, and S genomic segments; *ACTB* (human beta actin mRNA control) \| \| ^3^ paired serology – C6 peptide EIA, *B. microti* EIA, *B. burgdorferi* IgG and IgM Western blot, *A. phagocytophilum*, *E. chaffeensis, E. muris* subsp. *eauclairensis*, spotted fever group *Rickettsia* and *B. mayonii* IFA \| | | | | | | |
|  | | | | | | |
